# The Effects of Switching to Video Therapy on In-Session Processes in Psychotherapy During the COVID-19 Pandemic

**DOI:** 10.1007/s10488-024-01361-7

**Published:** 2024-03-14

**Authors:** Susanne Edelbluth, Brian Schwartz, Wolfgang Lutz

**Affiliations:** Department of Clinical Psychology and Psychotherapy, University of Trier, 54286 Trier, Germany

**Keywords:** face-to-face treatment, COVID-19, Therapeutic alliance, Coping skills, Emotional involvement, Piecewise regression, Multilevel modeling

## Abstract

**Objective and Aim:**

This study aimed to assess the impact of switching from face-to-face (f2f) psychotherapy to video therapy (VT) due to the COVID-19 pandemic on in-session processes, i.e., the therapeutic alliance, coping skills, and emotional involvement, as rated by both patients and therapists.

**Methods:**

A total of *N* = 454 patients with mood or anxiety disorders were examined. The intervention group (IG) consisted of *n* = 227 patient-therapist dyads, who switched from f2f to VT, while the control group (CG) consisted of *n* = 227 patient-therapist dyads, who were treated f2f before the pandemic. To evaluate the effects of switching to VT on in-session processes, three longitudinal piecewise multilevel models, one per process variable, were fitted. Each process variable was regressed on the session number with a slope for the three sessions before switching to VT and a second slope for up to six VT sessions afterwards.

**Results:**

The therapeutic alliance significantly increased after switching from f2f to VT across the two groups (IG and CG) and raters (patients and therapists) with no differences between IG and CG. On average, patients rated the therapeutic alliance better than therapists. Coping skills significantly increased after switching from f2f to VT across the two groups and raters, but the CG rated coping skills higher than the IG after the switch. Overall, therapists rated coping skills higher than patients. Emotional involvement did not significantly increase after switching to VT across the two groups and raters and there was no significant difference between patient and therapist ratings.

**Discussion:**

In conclusion, the switch to VT had no negative impact on the therapeutic alliance and emotional involvement. However, more coping skills were reported in the CG than in the IG after the switch to VT, which was mainly due to a stagnation in patient-rated coping skills in the IG.

**Supplementary Information:**

The online version contains supplementary material available at 10.1007/s10488-024-01361-7.

## Introduction

Due to the COVID-19 pandemic, social contacts had to be restricted worldwide to interrupt infection chains. Obviously, this also affected direct contacts between psychotherapists and their patients. For this reason, many psychotherapies were forced to abruptly switch from face-to-face (f2f) to video therapy (VT). There were concerns that this switch in modality could cause a rupture in the therapeutic alliance (Békés et al., 2021), make the recognition of emotions more difficult due to the physical distance (Aafjes-van Doorn et al., 2020; Markowitz et al., 2021; Thompson-de Benoit & Kramer, 2021), and impair the development of coping skills (Buckman et al., 2021), thereby negatively affecting therapy outcomes. However, empirical evidence of the impact of this forced switch to VT on therapeutic processes is lacking. There has been an increase in research on processes in psychotherapy in recent years, which aims to identify the processes, mechanisms, and strategies relevant for change in psychological therapy (e.g., Kazantzis et al., 2018; Lutz et al., 2023). According to Grawe’s (1997) theoretical framework, general change mechanisms include the therapeutic alliance, mastery/coping, clarification of meaning, problem activation, and resource activation. Based on this work, Rubel et al. (2017) examined the within- and between-patient effects of the therapeutic alliance, coping skills, and emotional involvement as in-session processes on symptom severity. The therapeutic alliance reflects patients’ perceived relationship quality with their therapists. Coping skills reflect patients perceived corrective experiences related to mastery/coping and clarification of meaning. Emotional involvement refers to the process of addressing patients’ problems in an emotionally responsive way. In this study, therapists also assessed all three in-session processes, i.e., rated the perceived quality of the relationship, the extent to which they worked on coping skills, and their patients’ emotional involvement. Research on process-outcome associations in f2f psychotherapy has shown a positive correlation between alliance and outcome (Horvath et al., 2011), significant effects of coping skills on outcome (Flückiger et al., 2013; Mander et al., 2013; Rubel et al., 2017), and also a positive association between emotional involvement in therapy and symptom improvement (Castonguay et al., 1996). In more detail Rubel and colleagues (2017) concluded that psychotherapy sessions are beneficial for patients if they learn new strategies to cope with problems: a positive therapeutic alliance or being emotionally involved in a session was not followed by symptom improvement in the next session if the patient was not also equipped with coping skills by the therapist.

The efficacy of VT in reducing symptoms has been demonstrated by meta-analyses and reviews, which have found no differences between VT and f2f psychotherapy in symptom reduction in randomized controlled trials (Batastini et al., 2021; Berryhill et al., 2019). First studies with naturalistic data have been able to replicate this finding in routine care, showing a per-session symptom reduction after switching from f2f to VT comparable to patients who were treated completely f2f (Lutz et al., 2021). To date, studies on in-session processes in VT mainly focused on the therapeutic alliance (Simpson & Reid, 2014; Simpson et al., 2021). It has been shown that a therapeutic alliance can be established in VT and that the therapeutic alliance does not differ between patients in f2f and VT (e.g., Norwood et al., 2018; Bouchard et al., 2000). However, therapists seem to rate the therapeutic alliance in VT lower than patients (Simpson, & Reid, 2014), and also lower than in f2f psychotherapy (Rees, & Stone, 2005). Initial studies on the therapeutic alliance and the switch from f2f to VT during the COVID-19 pandemic have demonstrated that there was no decrease in patient- and therapist-rated alliance due to the switch (Eichenberg et al., 2022).

Going beyond the alliance, one study investigated all general change mechanisms according to Grawe in an internet-based cognitive behavioral intervention for family caregivers (Theurer et al., 2021). They found that treatment outcomes were associated with therapists’ overall experiences of alliance, clarification, and mastery, that participants experienced more problem actuation than their therapists perceived, and that only participants’ and therapists’ experiences of clarification differed over time. Research asking analytic therapists about their experiences of switching to remote therapy and comparing it to their previous f2f sessions concluded that, despite technical and relational challenges, they felt just as strong, emotionally connected, and authentic in their online sessions as they did in the f2f sessions (Békés et al., 2020). However, approximately one-third of therapists reported that they experienced difficulty in connecting emotionally with their patients and reading patients’ emotions. Two-thirds of therapists also reported that the therapeutic alliance felt just as strong in remote therapy as in previous f2f sessions and half of the therapists asked felt just as emotionally connected to their patients (Bekes et al., 2020). Another study found that therapists, who identified more with a cognitive-behavioral approach, had a more positive attitude towards online therapy than psychodynamic therapists (Bekes, & Aafjes- van Doorn, 2020): the authors concluded that in psychodynamic therapies the focus is on the relational processes and non-verbal communication during the session and that these subtle processes may be more difficult to capture via videoconferencing. Furthermore, therapists reported challenges with remote therapy, such as limited perceptual awareness, technical problems, and signs of being tired (Stadler et al., 2023). Moreover, therapists also described differences in the therapeutic interventions used, with more than a third of therapists stating that they used fewer or adapted interventions in VT compared to f2f therapy (Stadler et al., 2023). The information on the intensity of the sessions and the development or maintenance of the therapeutic alliance was ambivalent: almost less than half of the therapists reported a stronger therapeutic alliance, but almost as many reported less closeness in the alliance during remote therapy (Stadler et al., 2023). In addition, 59% of therapists reported a lower intensity of psychotherapeutic work in remote therapy than in f2f (Stadler et al., 2023). Probst and colleagues (2021) asked patients and therapists about the differences in in-person and remote therapy in psychodynamic, common-factors, person-centered, process-experiential, interpersonal, cognitive, behavioral, and dialectical-behavioural interventions. The results showed that therapists rated all categories as more typical for f2f than for remote treatment. Patients rated all scales, except interpersonal therapy, higher for f2f than for remote therapy, but only the differences for psychodynamic, process-experiential, and cognitive interventions were significant (Probst et al., 2021). In addition, patients felt more confident and less intimidated to talk about their emotional status and problems in VT (Simpson, & Reid, 2014), thus Russel (2021) referred to the disinhibiting effect in online setting, which leads to opening up more in VT or telephone settings.

To our knowledge, there are no studies that have examined coping skills and emotional involvement in VT or while switching from f2f to VT immediately after each session. The present study aimed to assess the impact of switching the treatment setting from f2f to VT on in-session processes in psychotherapy (i.e., therapeutic alliance, coping skills, and emotional involvement) rated by both patients and therapists. To expand the findings on the impacts of switching to VT, the following research questions were addressed in the present study in patients and therapists who experienced a forced switch of treatment modality from f2f to VT in their psychotherapy during the COVID-19 pandemic:

Research question 1: Do the ratings of the therapeutic alliance, coping skills and emotional involvement differ between the IG (who had to switch from f2f to VT) and the CG (who had no switch in treatment setting)?

Research question 2: Do patients and therapists differ in their ratings in therapeutic alliance, coping skills, and emotional involvement?

Considering the summarized findings on the therapeutic alliance in the context of VT, we assume that therapists rate the therapeutic alliance lower than patients and that the switch from f2f to VT has no negative impact on the therapeutic alliance. In addition, we assume that coping skills and emotional involvement are rated lower by therapists in the IG than in the CG. We expect that patients are more likely to rate coping skills lower in VT than in f2f and emotional involvement at least as high in VT as in f2f, and that the patients’ and therapists’ ratings also differ in the two in-session process variables.

## Method

At the onset of the COVID-19-pandemic, *N* = 484 patients were currently receiving integrative cognitive behavioral therapy at an outpatient clinic in southwest Germany. To be included in this study, patients had to have received treatment for at least three sessions, be diagnosed with an affective or anxiety disorder, and switch from f2f to VT within a two-week period (starting from March 18, 2020), meaning that those patients had not experienced a longer interruption in treatment. The outpatient clinic had an existing infrastructure, in which every therapy room was fully equipped with computers and processes as well as outcome were routinely monitoring via the Trier Treatment Navigator (TTN; Lutz et al., 2019, 2022), a comprehensive feedback and decision-support system enabling data-informed psychological treatment. Therefore, patients and therapists were able to switch to video therapy quickly and without a longer interruption, and routine data collection was able to be continued online without any loss of data.

Diagnoses were based on structured clinical interviews for the *Diagnostic and Statistical Manual of Mental Disorders, fourth version* (SCID-I; First et al., 1995). Of the *N* = 484 patients, *n* = 84 ended therapy because of the onset of the pandemic, *n* = 33 patients paused therapy because of the onset of the pandemic, and *n* = 96 patients had sessions via telephone after the onset of the pandemic. Of the remaining *n* = 271 patients, *n* = 21 were excluded because of not having a mood or anxiety disorder and *n* = 23 patients were excluded because they had less than three sessions before the switch to VT. The remaining *n* = 227 patients were examined within the intervention group (IG). As a control group (CG) *n* = 227 patients from an archival dataset were propensity score matched using nearest neighbor one-to-one matching. This one-to-one matching method was based on the IG patients’ symptom improvement during their last three sessions before the switch. They were matched with archival cases with the most similar change in the respective sessions. The archival dataset consisted of *N* = 3415 patients who had completed their treatment at the outpatient clinic before the onset of the COVID-19-pandemic (see also Lutz et al., 2021).

### Treatment

In both the IG and CG, therapies were conducted at a university outpatient training and research clinic in Southwest Germany. At the outpatient clinic, process and outcome data were routinely collected for the purpose of supervision and research. All therapists had a Master’s degree in clinical psychology and were currently participating in a 3–5-year postgraduate psychotherapy training program with an integrative cognitive behavioral therapy focus. Trainee therapists had at least 1.5 years of clinical experience prior to treat patients at the outpatient clinic. Therapists were supervised by a senior therapist every fourth session. For a more detailed description of the training program, see Uhl et al. (2022). In addition, therapists were supported by a feedback system, the TTN (Lutz et al., 2019), which monitored patient outcomes and in-session process variables. Psychometric feedback was provided to therapists after each session. No therapists in the IG had ever provided remote therapy, e.g., via video, before. Patients and therapists gave their consent to use data for scientific research (psychometric and sociodemographic data) after being provided with verbal and written information. Therapy sessions lasted 50 min and took place weekly.

### Sample Characteristics

#### IG Patients

Most IG patients (64.8%; *n* = 147) were female, an average of *M* = 31.12 years old (*SD* = 11.58) and born in Germany (94.3%; *n* = 214). 54.2% (*n* = 123) of patients were in a committed partnership and 62.1% had a general qualification for university entrance (*n* = 141). Patients in the IG had an average of *M* = 2.09 (*SD* = 1.00) diagnoses with a range of 1 to 5. Examining all diagnoses, 79.7% (*n* = 181) of IG patients had an affective and 27.8% (*n* = 63) an anxiety disorder. As the primary diagnosis, most patients had an affective disorder (53.7%; *n* = 122), 11.5% (*n* = 26) had an anxiety disorder, 20.7% (*n* = 47) had a stress or adjustment disorder, and 14.1% (*n* = 32) were diagnosed with other disorders.

#### CG Patients

Most CG patients (57.3%; *n* = 130) were female, an average of 36.72 years old (*SD* = 12.92) and born in Germany (95.6%; *n* = 217). 58.5% (*n* = 133) of patients were in a committed partnership and 45.4% had a general qualification for university entrance (*n* = 103). Patients in the CG had an average of *M* = 2.67 (*SD* = 1.13) diagnoses with a range of 1 to 5. Examining all diagnoses, 83.7% (*n* = 190) of CG patients had an affective and 47.6% (*n* = 108) an anxiety disorder. As the primary diagnosis, most patients had an affective disorder (56.4%; *n* = 128), 15.9% (*n* = 36) had an anxiety disorder, 18.1% (*n* = 41) had a stress or adjustment disorder, and 9.6% (*n* = 22) were diagnosed with other disorders.

#### IG Therapists

IG therapists (*n* = 53) were an average of 29.50 (*SD* = 4.91) years old, 62.3% (*n* = 33) were female and 96.2% (*n* = 51) were German. Therapists treated an average of 5.87 (*SD* = 2.72) patients, with a range of 1 to 11.

#### CG Therapists

CG therapists (*n* = 128) were an average of 30.73 (*SD* = 6.16) years old, 54.7% (*n* = 70) were female and 95.3% (*n* = 122) were German. Therapists treated an average of 2.41 (*SD* = 1.43) patients, with a range of 1 to 7.

### Measures

To assess patients’ and therapists’ therapeutic alliance, coping skills, and emotional involvement ratings, the German version of the Bern Post Session Report (BPSR; Flückiger et al., 2010) was used. The BPSR is a self-report measure assessing patients’ and therapists’ subjectively perceived realization of process variables during the session. At the end of each session, patients filled out a 12-item and therapists an eight-item short form of the BPSR. All items are based on a 7-point Likert scale ranging from − 3 (“not at all”) to + 3 (“very strong”). Both versions showed good internal consistencies (for patients: α between 0.87 and 0.92; for therapists: α between 0.84 and 0.87) and have been validated and assessed in several previous studies (e.g., Flückiger et al., 2013; Rubel et al., 2017). The subscale *therapeutic alliance* consisted of four patient items and three therapist items. The subscale *coping skills* consisted of six patient items and three therapist items. The subscale *emotional involvement* consisted of two patient items and one therapist item (see Rubel et al., 2017). The exact items are provided in the appendix.

In addition, patients, who had to switch to VT, were asked about their distress due to the COVID-19 pandemic (“How are you feeling about the COVID-19 pandemic?“) on a visual analog scale from 0 (“Everything is fine”) to 100 (“It is a nightmare”). They were also asked about their well-being due to the pandemic (“Compared to how I felt before the COVID-19 pandemic, my general well-being is …”) on a Likert scale from 1 (“much better”) to 5 (“much worse”). Furthermore, the VT patients were asked to assess the perceived differences in the in-session process variables. They were asked to rate this for the therapeutic alliance (“I have the feeling that the relationship with my therapist has changed as a result of VT”), coping skills (“I have the feeling that my therapist does not understand my problems in VT as well as in f2f therapy”) and emotional involvement (“I am just as honest in VT and can show my feelings just as honestly as in f2f therapy”) on a Likert scale from − 3 (“not at all”) to + 3 (“very much”).

### Data Analysis

All analyses were conducted with the free software environment R version 4.0.1 (R Core Team, 2020). Multilevel analyses were conducted using the R packages lme4 v1.1–23 (Bates et al., 2015) and lmerTest v3.1–2 (Kuznetsova et al., 2017). Visualizations were displayed using the R package ggplot2 v2.2.0 (Wickham, & Wickham, 2016).

For CG session selection, each matched CG patient was assigned the session number of the switch from f2f to VT in the corresponding IG patient. The three sessions before this fictious switch session and the six sessions afterwards were included in the data analyses of the CG.

To evaluate the effect of switching from f2f to VT, a piecewise multilevel model with sessions nested within patient-therapist dyads was fitted for each of the three process variables. Each model was fitted with a slope for the three sessions before switching to VT and a slope for up to six sessions afterwards by regressing the respective process variable on the session (–3, − 2, − 1, 0, 1, …). Condition (switch to VT [IG] vs. no switch to VT [CG]) and raters (patients vs. therapists) were added as potential level 2 predictors of the intercept and slope of the growth curves. Effects were adjusted for the session number of the switch. In the random-intercept random-slope models, the intercepts of the growth curves and their slopes were allowed to vary between patient-therapist dyads. The data was analyzed within a hierarchical (two-level) structure with sessions nested within patients. Although patients were nested within therapists, we did not account for therapist effects and decided not to use the three-level structure for two reasons: First, many therapists, especially in the CG, had treated only one patient. Second, a recent simulation study suggested that in longitudinal models, including the third level (therapists) might decrease model fit (Falkenström et al., 2022). Missing values were not imputed since multilevel models can handle missing data well. Patient-therapist dyads were included in the IG analysis when at least three f2f sessions were conducted before the onset of the COVID-19 pandemic and the forced switch to VT and at least one VT session was conducted afterward. Moderating condition and rater effects were further visualized via simple slope analyses. In a sensitivity analysis, first the sociodemographic variables of the patients and therapists were tested on significant differences between the IG and the CG using χ^2^- or t-tests and second the significant differences found in the characteristics of the patients and therapists were included as potential predictors in each of the three models of the in-session process variables to adjust for these baseline differences. In an additional sensitivity analysis, the moderating effects of distress and well-being due to the pandemic on each process variable in the VT patients were evaluated. Each model was adjusted for the indicated change in the process variable as assessed by the patients and for the switching session. For *n* = 138 patients who switched to VT, those data were available.

## Results

### Main Analyses

**Fig. 1:**
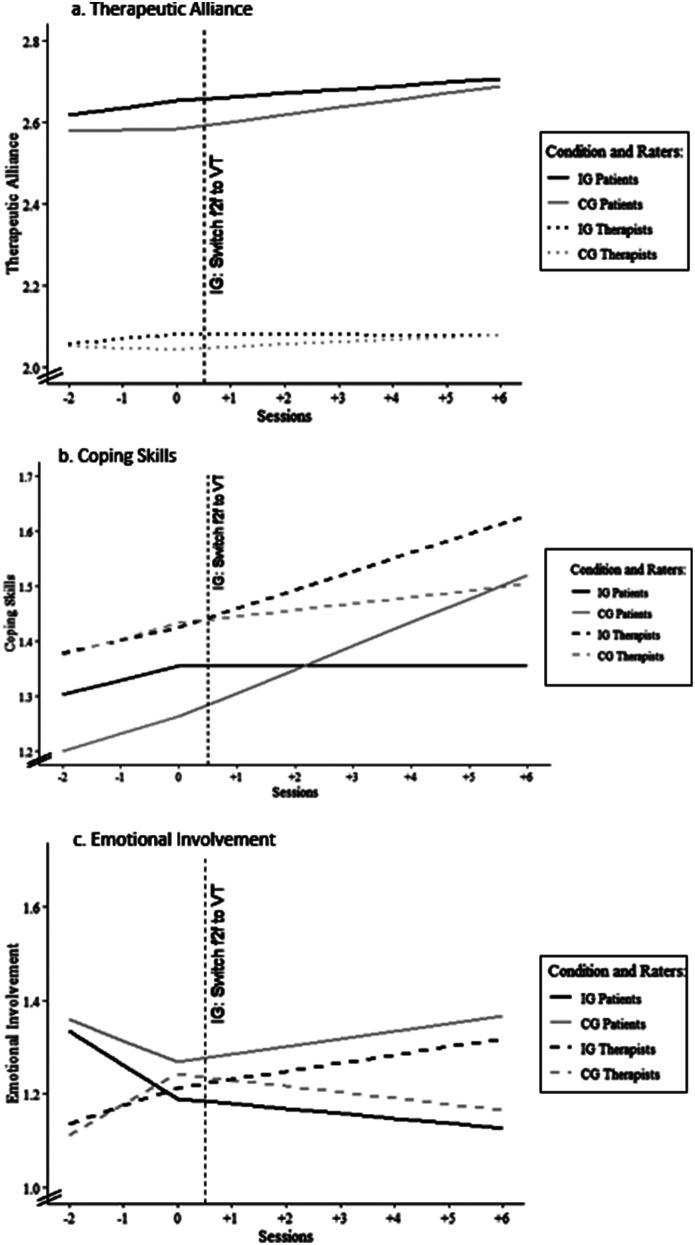
Longitudinal piecewise models for f2f and VT for (**a**) therapeutic alliance, (**b**) coping skills and (**c**) emotional involvement. *Note* Condition = IG vs. CG; Raters = Patients vs. Therapists; IG = Intervention Group with patients and therapists who switched from f2f to VT; CG = Control Group; f2f = face-to-face; VT = Video Therapy. Session − 2 to 0 in IG and CG f2f. Sessions 1 to 6 in IG VT and in CG f2f. Notice that different y-axis intercepts have been used in the graphs for better visualization

*Therapeutic alliance.* There was a significant increase in therapeutic alliance after the switch from f2f to VT across condition and rater (*b* = 0.017, *t* = 2.587, *p* < .001), whereby the IG and the CG did not differ significantly (*b* = − 0.008, *t* = − 0.873, *p* = .383). A significant difference between patient and therapist ratings was found (*b* = − 0.54, *t* = − 18.769, *p* < .001), in which patients rated the therapeutic alliance better than therapists. Furthermore, a significant effect of the switching session was identified (*b* = 0.009, *t* = 7.219, *p* < .001), meaning that the therapeutic alliance was rated higher in later switching sessions. There were no other significant main effects or interactions. For more details, see Table 1. For a graphic illustration, see Fig. 1.

**Table 1:** Longitudinal piecewise multilevel models for in-session processes

		Therapeutic Alliance			Coping Skills			Emotional Involvement	
*Fixed Effects*		*Coefficient (SE)*	*p*		*Coefficient (SE)*	*p*		*Coefficient (SE)*	*p*
Intercept		2.584 (0.036)	< 0.001		1.263 (0.051)	< 0.001		1.269 (0.064)	< 0.001
Switching Session		0.009 (0.001)	< 0.001		0.009 (0.002)	< 0.001		0.008 (0.002)	< 0.001
IG vs. CG^†^		0.069 (0.050)	0.167		0.092 (0.071)	0.196		–0.079 (0.088)	0.369
Patient vs. Therapist^‡^		–0.54 (0.029)	< 0.001		0.170 (0.042)	< 0.001		–0.027 (0.060)	0.656
Last three f2f sessions		0.002 (0.018)	0.915		0.032 (0.026)	0.232		–0.045 (0.038)	0.243
First six VT sessions		0.017 (0.007)	0.010		0.043 (0.010)	< 0.001		0.016 (0.014)	0.252
IG vs. CG^†^ * Slope 1		0.015 (0.022)	0.487		–0.005 (0.031)	0.859		–0.117 (0.045)	< 0.05
IG vs. CG^†^ * Slope 2		–0.008 (0.010)	0.383		–0.043 (0.013)	< 0.01		–0.027 (0.020)	0.179
Patient vs. Therapist^‡^ * Slope 1		–0.005 (0.021)	0.809		–0.003 (0.031)	0.928		0.101 (0.044)	< 0.05
Patient vs. Therapist^‡^ * Slope 2		–0.012 (0.009)	0.177		–0.031 (0.013)	< 0.05		–0.029 (0.018)	0.109
IG vs. CG^†^ * Patient vs. Therapist^‡^		–0.032 (0.035)	0.360		–0.099 (0.051)	< 0.05		0.049 (0.072)	0.497
IG vs. CG^†^ * Patient vs. Therapist^‡^ * Slope 2		0.002 (0.011)	0.864		0.065 (0.017)	< 0.001		0.057 (0.024)	< 0.05
*Random Effects*		*Variance*	*SD*		*Variance*	*SD*		*Variance*	*SD*
Intercept		0.185	0.431		0.371	0.609		0.452	0.672
Last three f2f sessions		0.002	0.041		0.001	0.037		0.012	0.110
First six VT sessions		0.001	0.030		0.001	0.029		0.004	0.061
Residual		0.270	0.520		0.570	0.755		1.171	1.082

*Note* IG = Intervention Group. CG = Control Group. f2f = face-to-face. VT = Video Therapy. ^†^Reference Group: IG. ^‡^Reference Group: Therapists. Slope 1 = Last three f2f sessions. Slope 2 = First six VT sessions

*Coping skills.* Overall coping skills significantly increased after the switch from f2f to VT across condition and rater (*b* = 0.043, *t* = 4.511, *p* < .001). In addition, the IG and the CG differed significantly in their increase in coping skills (*b* = − 0.043, *t* = − 3.198, *p* = .001) after the switch to VT, with the CG reporting a greater increase in coping skills than the IG. A significant difference was found between patient and therapist ratings over the sessions (*b* = 0.170, *t* = 4.066, *p* < .001), in which the therapists rated coping skills higher than patients. Furthermore, patient and therapist ratings of the last six sessions differed significantly (*b* = − 0.031, *t* = − 2.459, *p* = .014), with therapists reporting less of an increase in coping skills than patients. Furthermore, a significant negative interaction was found between condition and raters (*b* = − 0.099, *t* = − 1.967, *p* = .049), in which IG therapists rated coping skills higher than CG therapists and CG patients rated coping skills higher than IG patients. The increase in coping skills was significantly higher in the six sessions after the switch for IG therapists than for CG patients (*b* = 0.065, *t* = 3.891, *p* < .001). Furthermore, a significant effect of the switching session was identified (*b* = 0.009, *t* = 5.570, *p* < .001), meaning that coping skills were rated higher in later switching sessions. There were no other significant main effects or interactions. For more details, see Table 1. For a graphic illustration, see Fig. 1.

*Emotional involvement.* IG patients and therapists rated emotional involvement in the three sessions before the switch significantly lower than CG patients and therapists (*b* = − 0.117, *t* = − 2.586, *p* = .010). Also, across both conditions, therapists rated emotional involvement in the three sessions before the switch significantly higher than patients (*b* = 0.110, *t* = 2.498, *p* = .013). In the significant three-way interaction between raters, the condition, and the slope of the six video sessions (*b* = 0.057, *t* = 2.383, *p* = .017), therapists’ assessment of emotional involvement increased in the six video sessions in the IG, but not in the CG, whereas IG patients’ assessment of emotional involvement decreased in the six video sessions, while CG patients’ assessment increased. Furthermore, a significant effect of switching session was identified (*b* = 0.008, *t* = 4.382, *p* < .001), meaning that emotional involvement was rated higher in later switching sessions. No other main effects or interactions were significant. For more details, see Table 1. For a graphic illustration, see Fig. 1.

### Sensitivity Analyses

*Sociodemographic variables.* Patients in the IG and the CG did not differ in gender (*χ*_*1*_^*2*^ = 2.676, *p* = .102), nationality (*χ*_*1*_^*2*^ = 0.412, *p* = .521) and partner situation (*χ*_*1*_^*2*^ = 4.273, *p* = .370). Therapists in the IG and CG did not differ in gender (*χ*_*1*_^*2*^ = 1.095, *p* = .295), but differed in age (*F*_*451*_ = 10.139, *t* = -2.339, *p* = .010) and in the number of patients treated in this study (*F*_*452*_ = 149.270, *t* = 16.938, *p* < .001). Patients differed in their age (*F*_*452*_ = 9.000, *t* = -4.860, *p* < .001) and in the number of comorbid disorders (*F*_*452*_ = 6.735, *t* = -5.712, *p* < .001). When adding the previously described significant differences in the characteristics of the patients and therapists in each of the three models, for all three process variables, the same patterns emerged both in the main effects and in the interactions as in the three main analyses.

*Therapeutic alliance.* Patients stated that the alliance had not changed (*b* = − 0.088 *t* = − 3.040, *p* = .003), and the therapeutic alliance over the course of the VT sessions remained stable (*b* = 0.014, *t* = 0.482, *p* = .631). In addition, no other significant main effects or interactions were found between distress (*b* = 0.000, *t* = 0.594, *p* = .554), well-being (*b* = − 0.003, *t* = − 0.291, *p* = .772), and the session-alliance association.

*Coping skills.* There was no significant impact of whether patients reported that therapists understood their problems in VT as good as in f2f on coping skills (*b* = − 0.019, *t* = − 0.271, *p* = .787). The distress–session interaction (*b* = 0.001, *t* = 1.863, *p* = .065) and the well-being–session interaction (*b* = − 0.029, *t* = − 1.921, *p* = .058) were not significant.

**Fig. 2:**
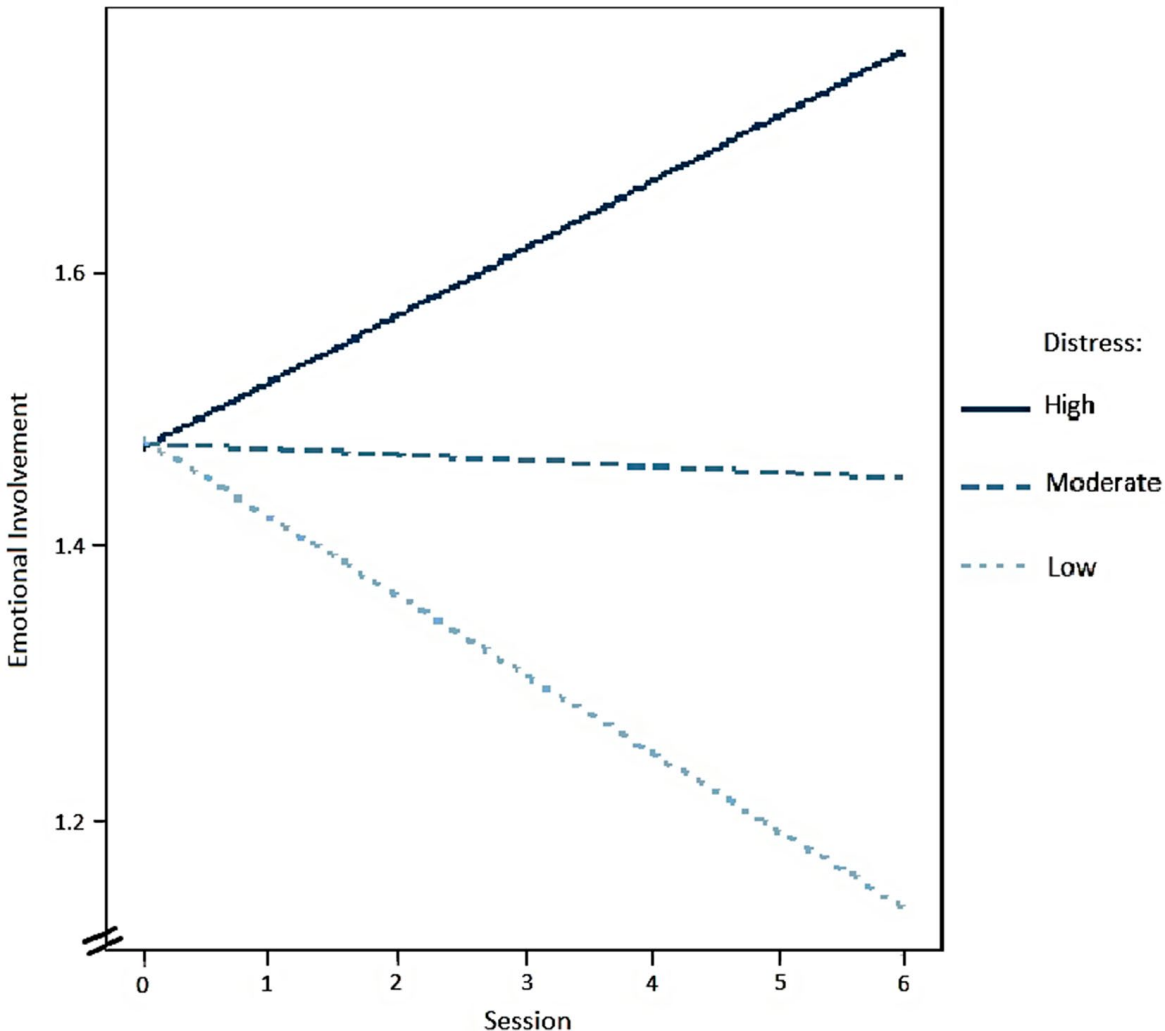
Moderating Effect between Distress because of the Pandemic and Emotional Involvement. *Note* Moderating Effect only for Patients in Intervention Group. Session 1 to 6 VT, Session 0 f2f

*Emotional involvement.* There was no significant impact of whether patients reported being as open and expressing their feelings in VT as in f2f (*b* = 0.076, *t* = 1.375, *p* = .172). The moderator analysis revealed that distress due to the pandemic significantly moderated the session − emotional involvement association (*b* = 0.002, *t* = 2.115, *p* = .037), patients whose distress was more influenced by the events related to the pandemic showed a stronger association between session and emotional involvement in VT. For a graphic illustration, see Fig. 2. All other main effects and the interaction between well-being due to the pandemic and session on emotional involvement (*b* = − 0.033, *t* = − 1.101, *p* = .274) were not significant.

## Discussion

In line with our hypotheses, therapist and patient ratings differed, as therapists rated the therapeutic alliance lower than patients and the switch from f2f to VT had no negative impact on the alliance. The hypotheses on coping skills were only partially confirmed: in line with our hypotheses, therapist and patient ratings differed and patients in the IG rated this process variable lower than in the CG. Contrary to our hypothesis, therapists rated this variable higher in the IG than in the CG and there was no difference between the IG and the CG. Contrary to our hypotheses, neither therapists and patients rated emotional involvement differently nor the two groups (IG vs. CG) differed. Consistent with our hypothesis, patients’ ratings did not differ between the two groups.

The results of this study suggest that the switch of treatment modality from f2f to VT seems to have no negative impact on an existing therapeutic alliance, as an increase in the alliance was found after the switch. On the contrary, the relationship was improved further and patients who switched to VT and the matched control cases had a comparable increase in the therapeutic alliance. However, therapists assessed the therapeutic alliance lower than patients did in both f2f and VT. Nevertheless, the therapeutic alliance was also perceived comparably between therapists who had to switch to VT and the matched control cases. Additionally, patients and therapists in both conditions reported no difference in their assessment of the therapeutic alliance over the course of treatment. The increase in the therapeutic alliance after switching treatment modality that Eichenberg et al. (2022) found was confirmed in this study. However, Eichenberg et al. (2022) only examined the therapeutic alliance as rated by patients. Our study extended this analysis to the therapists’ assessment of the therapeutic alliance. The significant difference in patient and therapist ratings of the therapeutic alliance over the course of the sessions is also consistent with previous findings that therapists rate the relationship lower than patients in VT (Simpson, & Reid, 2014) as well as generally in psychological treatments (Atzil-Slonim et al., 2015).

Furthermore, we found that coping skills ratings increased significantly across raters and condition over the course of the last six sessions. Over the course of the entire nine sessions and the condition, therapists rated coping skills higher than patients. However, a significant interaction was found between raters and change in coping skills during the first six sessions after the switch across condition, in which therapists rated coping skills lower than patients. In addition, a significant interaction was found between the condition and the slope of the last six sessions across raters, in which the patient-therapist dyads, who had to switch to VT, rated coping skills lower than the dyads who did not have a switch of setting. For the patients who switched to VT, neither an increase nor a decrease in coping skills were found in the video-based sessions. The IG therapists specifically tried to work more on coping skills with their patients in the video-based sessions, as an increase in this process variable was found. However, both effects described previously could also be interpreted as pandemic-related and not necessarily as a consequence of switching to VT. Patients who experienced the pandemic as stressful also showed a lower symptom reduction in VT than patients who dealt better with the effects of the pandemic (Lutz et al., 2021).

For emotional involvement, there were no significant differences between therapists and patients, or between the IG and the CG. Furthermore, there was no significant increase in emotional involvement across raters and conditions after switching to VT. This could be explained by the *online calming hypothesis* (Reynolds et al., 2013), which assumes that therapists and patients perceive the online environment as more comfortable and less threatening than f2f contact. A lower level of arousal in VT than in f2f contact has been shown for therapists and patients with anxiety disorders and depression (Reynolds et al., 2013). Simpson and colleagues (2021) concluded that the additional safety that can be experienced in VT makes it a less threatening option, especially for anxiety disorders. Patients who had to switch to VT showed a non-significant decrease in emotional involvement over the course of the video-based sessions. The therapists who had to switch to VT reported an increase in emotional involvement over the course of the video-based sessions, meaning that the therapists specifically worked towards getting their patients more emotionally involved in these sessions. However, as the emotional involvement scale consisted of only one item for therapists and two items for patients, the results of this study should be interpreted with caution regarding this in-session process variable for the impact of switching to VT. Moreover, at the time the assessments were conducted (shortly before and during the first weeks of the lockdown), it was probably more important for IG therapists to work on coping skills than on emotional involvement. Therapists may have done this intuitively in order not to emotionally overburden or overwhelm patients even more.

The sensitivity analyses showed no moderating effects of the impact of the pandemic on the course of the therapeutic alliance and coping skills. However, for the course of emotional involvement, there was a moderating effect of the impact of the pandemic in VT patients. Patients whose distress was more influenced by the pandemic showed a stronger increase in emotional involvement in the VT sessions.

### Limitations, Strengths, and Future Directions

This study has some limitations. The relatively small and selective sample of *n* = 227 patients is the main limitation. This means that not every patient in the outpatient clinic switched treatment when f2f treatment was no longer possible. In total, *n* = 117 patients decided to pause their treatment for a while, and of these, *n* = 84 patients decided to end their treatment sometime later. The specific reasons for their decision are not known. Some might have been less impaired at that time and might have thought that the lockdown and the pandemic would not last that long. Others may have been afraid to use VT tools or did not have the technical requirements for VT. Another important limitation of this study that should be considered is that only patients with mood or anxiety disorders were analyzed and therefore the results cannot be generalized to other disorders. For example, we cannot guarantee that patients with a personality disorder will not experience a disruption in the therapeutic alliance due to a setting switch. Another limitation refers to data collection in the CG. The CG was not collected simultaneously with the IG during the COVID-19 pandemic, as these are naturalistic data and f2f therapy was not possible at this time due to the lockdown. This is why the CG was matched from an archival dataset. Additionally, therapists and patients differed in their age, for which we controlled in a sensitivity analyses. However, no differences were found in the patterns of the main effects and the interactions in those analyses and the main analyses. In another sensitivity analyses we attempted to control for the impact of the pandemic on the in-session process variables. We could not find any moderating effects for the therapeutic alliance and coping skills, but for emotional involvement.

Despite these limitations, this study underlines VT as an important alternative treatment modality in psychotherapy, taking into account multiple patient- and therapist-rated in-session process variables over the course of several sessions. Nevertheless, future studies should expand on the present study’s findings. Not only the short-term effects of switching treatment form should be examined, but also the long-term effects on therapy outcome, in particular, the impact of in-session process variables in VT on therapy outcome. Furthermore, it would also be important to examine these variables under non-pandemic circumstances in VT, as a confounding effect of the previously unprecedented burden cannot be completely excluded.

## Conclusions

Overall, the results of the study suggest that VT can be an alternative to f2f therapy, especially when a switch of setting is necessary due to external circumstances. There was no difference between the IG and CG over the course of the sessions and across raters for all three in-session variables. Especially switching to VT had no negative impact on the therapeutic alliance for both patients and therapists. However, therapists should consider patients’ potential concerns and difficulties regarding VT and ideally discuss them beforehand. In addition, it might be useful to integrate psychotherapy via VT into psychotherapy training programs to reduce therapist concerns and difficulties regarding VT. Further research is needed to confirm these findings and identify the mechanisms underlying the effects of VT on therapeutic outcomes. More specifically, the in-session process variables should be examined in VT under non-pandemic conditions.

### Electronic Supplementary Material

Below is the link to the electronic supplementary material.

Supplementary Material 1
