# Supplementary material for: The Effects of Switching to Video Therapy on In-Session Processes in Psychotherapy During the COVID-19 Pandemic
Source: Adm Policy Ment Health. 2024 Mar 14;51(4):428–38. doi: 10.1007/s10488-024-01361-7 (PMC11196328; doi:10.1007/s10488-024-01361-7)
Supplement: Supplementary file 1 — Supplementary Material 1 [file 10488_2024_1361_MOESM1_ESM.docx]

**Appendix**

The Therapeutic Alliance scale is comprised of four items for the patient’s version:

- Today I felt comfortable with my therapist.
- The therapist and I are getting along well.
- I believe that the therapist is truly interested in my wellbeing.
- I feel that the therapist has real appreciation for me.

The Therapeutic Alliance scale is comprised of three items for the therapist’s version:

- Today I felt comfortable with my patient.
- The patient and I are getting along well.
- The patient and I work on shared goals.

The Coping Skills scale is comprised of six items for the patient’s version:

- Now I feel better up to situations, to which I have not felt up to until now.
- Now I’m more confident in my ability to solve problems by myself.
- I have the feeling that I better understand myself and my problems.
- Today we got closer to the core of my problems.
- Today I became aware why I react towards some people in a certain way and not in a different way.
- Now I know better what I want.

The Coping Skills scale is comprised of three items for the therapist’s version:

- Today I have worked towards the patient being able to cope better with situations that are difficult for him.
- Today I specifically tried to improve the patient's ability to act.
- Today I actively worked towards the patient being able to see his problems in new contexts.

The Emotional Involvement scale is comprised of two items for the patient’s version:

- Today I was involved emotionally in an intensive way.
- What we talked about today affected me.

The Emotional Involvement scale is comprised of one item for the therapist’s version:

- Today, I worked specifically to engage the patient emotional
